# An Auto-Focus Method of Microscope for the Surface Structure of Transparent Materials under Transmission Illumination

**DOI:** 10.3390/s21072487

**Published:** 2021-04-02

**Authors:** Yang Liao, Yonghua Xiong, Yunhong Yang

**Affiliations:** 1 State Key Laboratory of High Field Laser Physics, Shanghai Institute of Optics and Fine Mechanics, Chinese Academy of Sciences, Shanghai 201800, China; superliao@siom.ac.cn; 2 School of Automation, China University of Geosciences, Wuhan 430074, China; yangyh@cug.edu.cn; 3 Hubei Key Laboratory of Advanced Control and Intelligent Automation for Complex Systems, Wuhan 430074, China

**Keywords:** auto-focus, microscope, transmission illumination, deep convolution neural network, differential evolution

## Abstract

This paper is concerned with auto-focus of microscopes for the surface structure of transparent materials under transmission illumination, where two distinct focus states appear in the focusing process and the focus position is located between the two states with the local minimum of sharpness. Please note that most existing results are derived for one focus state with the global maximum value of sharpness, they cannot provide a feasible solution to this particular problem. In this paper, an auto-focus method is developed for such a specific situation with two focus states. Firstly, a focus state recognition model, which is essentially an image classification model based on a deep convolution neural network, is established to identify the focus states of the microscopy system. Then, an endpoint search algorithm which is an evolutionary algorithm based on differential evolution is designed to obtain the positions of the two endpoints of the region where the real focus position is located, by updating the parameters according to the focus states. At last, a region search algorithm is devised to locate the focus position. The experimental results show that our method can achieve auto-focus rapidly and accurately for such a specific situation with two focus states.

## 1. Introduction

In the field of materials science, the laser is widely used to micromachine transparent materials, including glass, crystal, polymer, etc. [1,2,3]. The auto-focus of the microscope is crucial for laser micromachining to ensure that the laser energy can automatically converge on the target point, where effective illumination is the basic environmental requirement. There are two common illumination modes for microscopy systems: reflection illumination and transmission illumination. For the reflection illumination, the light sources are placed between the object to be observed and the camera system, which is conducive to observing the surface of materials, especially for opaque materials. For the transmission illumination, the light sources are placed behind the object to be observed. Due to the different positions of light sources, compared with reflection illumination, transmission illumination is more suitable to observe the interior of transparent materials. To achieve efficient laser micromachining of transparent materials from surface to interior, it is necessary to avoid the continuous switching of two illumination modes that interrupts micromachining and readjusts light parameters. Therefore, in many applications, transmission illumination is of certain significance to auto-focus of microscope for surface structure of transparent materials.

The existing auto-focus methods of the microscope can be categorized into active ones or passive ones [4]. Active auto-focus methods need the support of optical equipment, which is expensive and difficult to design [5]. Passive auto-focus methods are also called auto-focus methods based on digital image processing. They obtain the current image information by image processing technology to determine whether the current state is defocusing or focusing and control the lens to move to the focus position by some search strategies. The depth from defocus (DFD) and the depth from focus (DFF) are two types of passive auto-focus methods.

The DFD generally only needs to collect a few frames of images for analysis and processing, and then use the established defocus mathematical model of the optical imaging system to achieve autofocus. The DFF is based on the search process. It needs to collect several images and iteratively search for the best value of image clarity evaluation to achieve focus. The DFD estimates the depth information by establishing the mathematical model of the optical imaging system, whose error is largely due to the fact that it is hard to achieve satisfactory modeling precision in theory [6,7,8]. On the contrary, owing to the advantages, such as being simple to implement and easy to design, DFF-based auto-focus methods of microscope have become a research hotspot.

A DFF-based auto-focus method mainly includes two key steps: (1) evaluating the sharpness of the image by applying a sharpness measurement algorithm and (2) finding the focus position by a focus search algorithm, where the curve of sharpness values of image collected by a charge-coupled device (CCD) camera versus lens position is called the focus curve. For image sharpness measurement algorithms, most of them are based on spatial analysis [9], frequency analysis [10], and pixel statistics. In particular, image sharpness measurements based on the spatial domain are widely used due to their simple calculation and good stability. For focus search algorithms, many results are derived based on hill-climbing search [11], Fibonacci search [12,13], evolutionary computation [14], and mathematical model [15,16].

**Figure 1:**
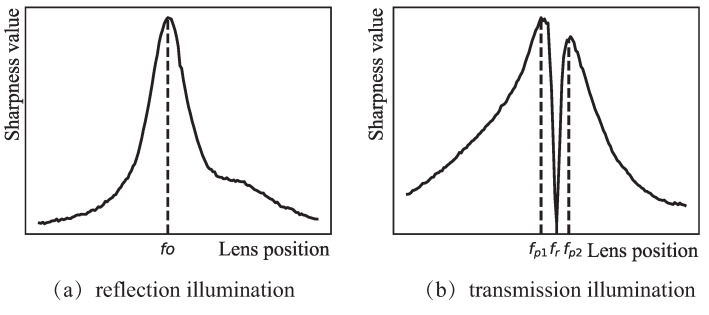
Focus curves under different illumination modes.

It should be mentioned that most existing DFF-based auto-focus methods are proposed for opaque materials as the observed objects, such as the embryonic cells of mouses, under reflection illumination. The resulting focus curves have a similar form to the one shown in Figure 1a, where the focus position fo can be found by the global maximum of sharpness in the focus curve. However, when using transmission light to illuminate the surface structure of transparent materials, due to the influence of optical imaging of particles on the surface, the shadow effect will be produced on the side far away from the light, and on the side closer to the light the brightness will be stronger. According to a large number of experimental observations, there may exist two different focus states at the lens positions $f_{p1}$ and $f_{p2}$ in the focus curve as shown in Figure 1b. The two lens positions $f_{p1}$, $f_{p2}$ can be regarded as two pseudo focus positions which mean two false focus points with two peaks of sharpness, while the real focus position $f_{r}$ is actually located in the region $\left[f_{p1},f_{p2}\right]$ with the local minimum value of sharpness. Obviously, this presents a totally different application situation that is beyond the capacity of the existing auto-focus methods of the microscope. To the best of our knowledge, such a phenomenon has received very little attention in the literature, even if it widely exists in applications such as femtosecond laser micromachining and biological imaging, and heavily requires the manual focus of the microscope. Thus, developing an effective auto-focus method for this special situation is necessary and has certain practical significance, which is the main motivation of the paper.

In this paper, a novel auto-focus approach is developed for the surface structure of transparent materials under transmission illumination, mainly including two steps: locating the two endpoints of the region $\left[f_{p1},f_{p2}\right]$ in Figure 1b at first, and then finding the real focus position in the region. The two-step optimization solution makes our approach different from the existing DFF-based auto-focus methods of microscope that only adopt one optimization search. The main contributions of this paper are summarized as follows.

1. A focus state recognition (FSR) model is established to judge the category of focus states from microscopic image, so as to effectively guide the adjustment of lens’ direction and moving range;
2. An endpoint search algorithm with memory and sorting (ESMS) is designed to find the two endpoint positions accurately and quickly. The ESMS effectively reduces the search time and the motor’s travel distance by introducing memory for the historical evaluation data and sorting for the population;
3. The mathematical relationship between the real focus position and the endpoint positions is disclosed by the correlation analysis and regression analysis of a large number of samples, such that the real focus position can be obtained by a region search algorithm.

The rest of this paper is organized as follows. Section 2 explains our auto-focus method in detail. The experimental results are demonstrated in Section 3 on slide glass to evaluate the effectiveness of our method and the conclusion is given in Section 4.

## 2. Main Results

**Figure 2:**
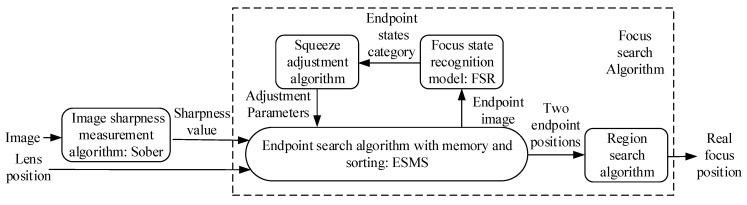
Overview of designed auto-focus method.

The overall architecture of our auto-focus method is illustrated in Figure 2. It is made up of several parts: an image sharpness measurement algorithm (The Sober operator), the FSR model, the ESMS, a squeeze adjustment algorithm, and a region search algorithm. The image collected from the CCD camera is calculated by the Sober operator to get the sharpness value, and the larger the average gray value of the image processed by the Sobel operator, the clearer the image. The sharpness value and the lens position are then input to the ESMS that approximates the two endpoints of the region where the real focus is located. The FSR model and the squeeze adjustment algorithm are combined to tune the parameters in the ESMS for the improvement in accuracy and convergence speed. Once the two endpoint positions are found, the region search algorithm then acts to get the real focus position. This method is a DFF-based auto-focus method, in which the Sober operator is used for image definition measurement, and another important part of the DFF-based auto-focus method is the focus search algorithm, which is composed of ESMS, FSR, squash adjustment, and region search.

In the following, each algorithm is explained in detail one-by-one.

### 2.1. Focus State Recognition Model

An image classification model based on a deep convolution neural network (DCNN) is designed to distinguish the focus states by the microscopic images. In this section, we discuss how to build a focus state recognition model.

**Figure 3:**
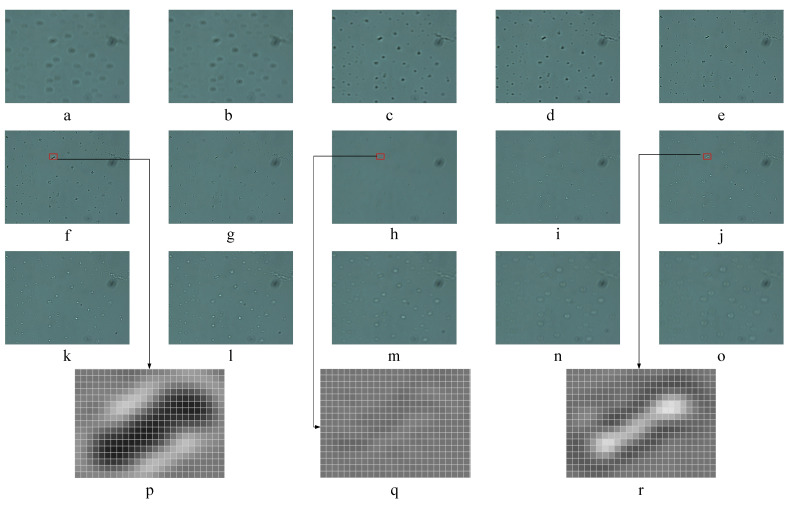
Microscopic image in focus process: (**a**–**o**) represent the sequence of microscopic images from defocused to focused and then to defocused in the focus process; (**h**) is the image that lens is at real focus position $f_{r}$. (**f**,**j**) are the images that lens is at two different pseudo focus positions $f_{p1}$, $f_{p2}$. (**p**,**q**,**r**) are locally enlarged pixel-wise gray image of (**f**,**h**,**j**) respectively.

A set of microscopic images of the focusing process is presented in Figure 3, and the position of the lens is during the movement from −80 μm to 80 μm. A set of microscopic images of the focusing process is presented in Figure 3. In particular, Figure 3h is the image showing that the lens is at the real focus position $f_{r}$. Figure 3f,j are the images showing that the lens is at two different pseudo focus positions $f_{p1}$ and $f_{p2}$. Figure 3p,q,r are locally enlarged pixel-wise gray images of Figure 3f,h,j, respectively. From Figure 3p–r, a change of brightness of the images can be observed. From Figure 3a–h, the pixel value is small and the color of image is dark, and this focus state is called dark state. From Figure 3h–o, the pixel value is grow large and the color of image is bright, and this focus state is called bright state. Figure 3h is the image showing the transitional point between two focus states. It can be seen that the image content is extremely sparse, but the texture is fine, and it is the imaging surface completed by focusing on the transparent material surface.

From Figure 3p,r, it can be seen that the closer to the pseudo focus position, the more obvious difference of local texture features. Through a large number of sample image analysis, combined with the related optical imaging analysis, it can be clear that the phenomenon of two focus states of the transparent material surface structure under the transmission light is caused by the influence of the light on the particles on the transparent material surface, and this phenomenon is widespread. Although the feature differences between the two focus states are easily observed by human eyes, it is difficult to design feature extractors manually. In view of this situation, DCNN can solve this problem well, and automatically mine the hidden feature differences in these images through the convolution layer of DCCN.

DCNN models deliver state-of-the-art performance for classification and recognition tasks, especially in the field of computer vision [17,18,19,20]. In recent years, a large number of DCNN architectures are proposed, such as VGGNet [21], GoogLeNet [22], ResNet [23], MobileNetV1-V3 [24,25,26] and ShuffleNet [27], as the basic networks of DCNNs, which are widely migrated to image classification model for feature extraction. Among them, the MobileNetV2 of the lightweight DCNN model MobileNet series is widely used in various industrial image classification scenarios due to its good robustness, small computation, and high accuracy. MobileNetV2 still uses the DepthWise convolution in MobileNetV1. The difference is that MobileNetV2 introduces the inverted residual structure and bottleneck layer: the inverted residual structure is used to increase the number of channels and obtain more features, and the output layer uses Linear instead of Relu to prevent Relu from destroying features. This new structure is called a bottleneck residual structure. Compared with the original structure, the improved method retains more information and improves the accuracy of the model.

**Figure 4:**
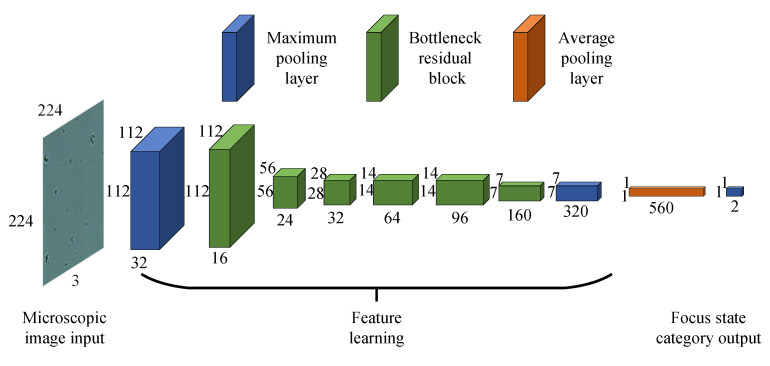
FSR architecture.

Inspired by MobileNetV2, an FSR model based on bottleneck residual block is established. The overall architecture of the established FSR is illustrated in Figure 4. In the FSR architecture, the green blocks represent bottleneck residual block and other color blocks represent standard convolution block. All standard convolution blocks use maximum pooling, except for orange blocks that use average pooling. The classification task for the focus state is a binary classification problem. FSR is a full convolution network. In the output layer, a sigmoid function is used to output a conditional probability for each class. Assuming that there are two classes of focus states for the input image *x*, its corresponding output probability $O_{j}\in \left[0,1\right]$ for class *j* is calculated as

(1) $$O_{j}=\frac{1}{1+e^{-(\theta (j)x)}},\;j=1,\;2$$

where θ is the model parameter to learn and $\sum_{j=1}^{2}O_{j}=1$. The output value of the corresponding dimension of the FSR model indicates the probability of the focus state to which it belongs. The first dimension and the second dimension of the output vector represent bright state and dark state, respectively.

### 2.2. Endpoint Search Algorithm with Memory and Sorting

The real focus position is the lens position with the lowest image measurement value in the region of the two pseudo focus positions by experimental analysis. Therefore, ESMS is designed to seek for the endpoint positions of the region $\left[f_{p1},f_{p2}\right]$, which means optimizing the lens positions when the image measurement value is the maximum in two different focus states. In essence, endpoint position search is a one-dimensional local extremum optimization problem. In addition, taking into account the randomness of the initial position of the lens, an ESMS is designed based on the differential evolution best (DE-best) algorithm [28,29]. The differential evolution (DE) is a population-based heuristic global search technology, having advantages in dealing with multi-extreme optimization problems. Compared with other evolutionary algorithms, DE has a simpler principle and fewer control parameters.

The DE-best emphasizes the convergence speed of the algorithm and meets the requirements of auto-focus for speed in the microscope, because it is feasible to use the positions near the pseudo focus as the endpoint positions. Therefore, premature phenomena of DE-best are allowed when a small number of populations and iterations are set. The objective function *J* of ESMS is as follows.

(2) $$J=\max_{P}F_{sober}\left(I\right)$$

The microscope lens position *P* is the optimization target, and the microscopic image acquired by the CCD camera at the position *P* is *I*. The fitness function $F_{sober}$ is the Sober operator to measure the sharpness of image *I*.

The sharpness value of the microscopic image is used as the fitness value of the individual in the population of ESMS. Each evaluation of the fitness function goes through the following three steps.

*Step* 1: move the microscope lens to the position *P*;
*Step* 2: obtain the microscopic image *I* of microscope lens at the position *P* by a CCD camera;
*Step* 3: evaluate the sharpness of microscope image I by using the Sobel operator image sharpness measurement algorithm.

Each evaluation of the fitness function of ESMS requires a focus evaluation operation, and Step 1 spends more time than the other two steps due to the limit of hardware devices. Therefore, in order to speed up the endpoint search, some measures should be taken to make the number of focus evaluation operations and the distance of the motor movement be reduced as much as possible. First, a data archive of historical search is established to make the algorithm have a memory function. The evaluation values and populations of each evaluation are saved to avoid repeated evaluations. Combined with the movement accuracy of the microscope lens, the evaluation of fitness function can be determined whether to perform focusing evaluation or directly read from the historical database. Then, a sorting strategy is added for standard DE-best to reduce the focus evaluation operation. Each individual of ESMS represents the position of the lens. All individuals of each generation population are sorted by a numerical value and move the lens in a certain sequence. On the one hand, it can reduce the total motion distance of the motor. On the other hand, it can avoid the damage to the motor system caused by repeatedly going back and forth.

Algorithm 1 shows the workflow of ESMS. The Mutation, Crossover and Selection functions in the ESMS are the same as the standard DE-best. The sort is a sort function. $H_{n}^{e,p}$ is the data vector of history evaluation of population, which saves lens position (*p*) and the evaluation value of the acquired image (*e*) of the individual (*n*).


**Algorithm 1:** ESMS algorithm

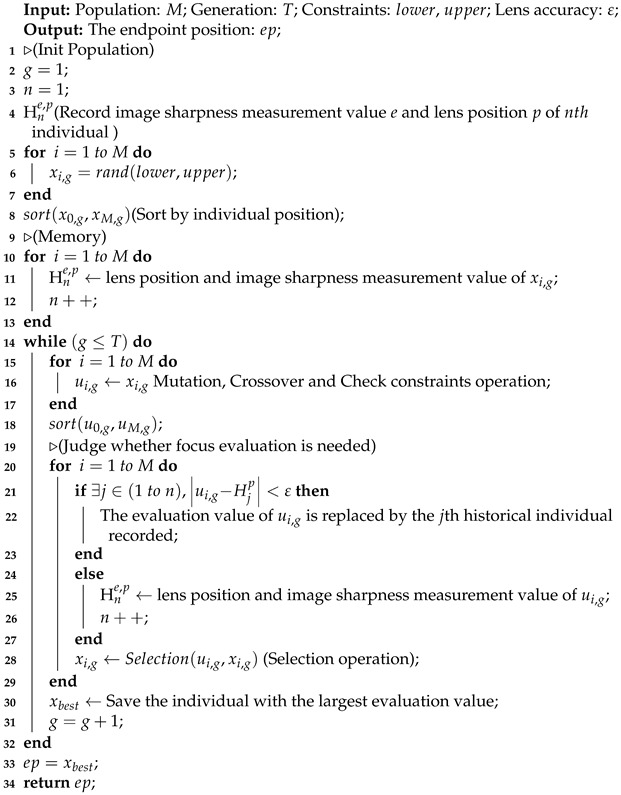




### 2.3. Squeeze Adjustment Algorithm

The purpose of the squeeze adjustment algorithm is to guide ESMS to adjust parameters. As shown in Algorithm 2, the squeeze adjustment algorithm gives the adjustment strategies of moving range and direction of the lens according to the category of the focus state and makes the search area of ESMS approach to the focus state which is different from the current focus state. Because the goal of ESMS is to optimize the lens positions in the peaks of image sharpness measurement value under different focus states. The squeezing adjustment algorithm is equivalent to increasing the attractive region of the effective peak and decreasing the attractive region of the invalid peak. When one of the endpoint positions is acquired, the success rate of ESMS searching another endpoint position is greatly improved by each execution of the squash adjustment algorithm. To limit the number of searches, the maximum number of search $T_{s}$ is set in Algorithm 2.
**Algorithm 2:** Squeeze adjustment algorithm
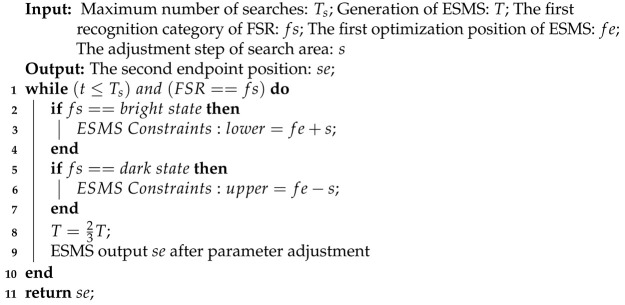


### 2.4. Region Search Algorithm

The region search algorithm describes how to get the real focus position from the two endpoint positions. Because the real focus position is the minimum value between two endpoint positions, the real focus position is obtained by traversal search. By the experimental analysis, it can be discovered that the distance between the two endpoint positions is only a dozen microns, far less than the total search distance. So it does not take a long time to use traversal search.

Besides, some definite mathematical relations between the pseudo focus positions and the real focus position are mined by a lot of experimental data analysis. Because the optimized endpoint positions are often pseudo focus positions, the endpoint positions can be used to predict the real focus position. This mathematical relationship between the pseudo focus positions and the real focus position is determined by the following methods.

The Pearson correlation is used to analyze the correlation between $f_{p12}$ and $f_{r}$. The sum of the two pseudo focus positions $f_{p12}$ and the Pearson correlation coefficient are given by (3) and (4), respectively.

(3) $$f_{p12}=f_{p1}+f_{p2}$$

(4) $$\rho_{XY}=\frac{Cov(X,Y)}{\sqrt{D(X)}\sqrt{D(Y)}}$$

where *D* is the variance function and Cov is the covariance function. The $\rho_{XY}\in \left[-1,1\right]$, and the larger the absolute value of $\rho_{XY}$, the stronger the linear correlation between *X* and *Y*. If $\rho_{XY}$ is higher than the linear correlation threshold commonly set to 0.9, it means that *X* and *Y* have a strong correlation. When the Pearson correlation coefficient between $f_{p12}$ and $f_{r}$ is higher than Lt, the traversal search or mathematical prediction can be selected to execute. If there is no obvious mathematical relationship between $f_{p12}$ and $f_{r}$, only traversal search can be selected. When the mathematical prediction is selected, a regression model is built as shown in (5).

(5) $$f_{r}=k\cdot f_{p12}+b$$

where *b* is generally limited to 0, so the position relationship between $f_{p12}$ and $f_{r}$ is obtained. According to the strength of the mathematical relationship between the real focus position and the pseudo focus positions, two regional search algorithms are given: traversal search and mathematical prediction.

## 3. Experiments and Analysis

**Figure 5:**
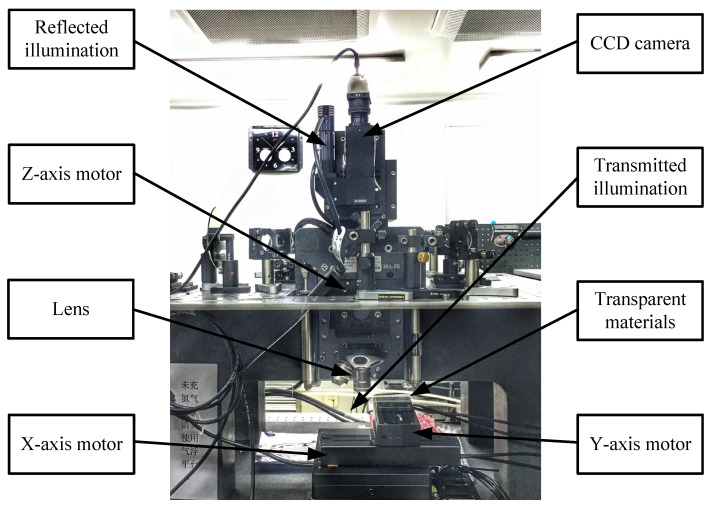
Microscope experimental platform.

A microscope experimental platform is shown in Figure 5, consisting of a three-axis electric system, a CDD camera, a group of lenses, two light sources, and transparent slide glass as test samples. The CCD camera has 80 W color pixels (i.e., 1024×768). The typical magnification of the lens is 20×. The z-axis motor (nano air flotation motion platform with minimum motion accuracy of 0.1 μm) is used to control the movement of the lens. The x-axis and y-axis motors are direct-current linear motors with minimum motion accuracy of 1 μm, and they are used to control the movement of the test samples.

In this section, several investigations are given on the effects of ESMS, training, and evaluation of the FSR model and performance of various region search algorithms, respectively.

### 3.1. Training and Evaluation of FSR Model

**Figure 6:**
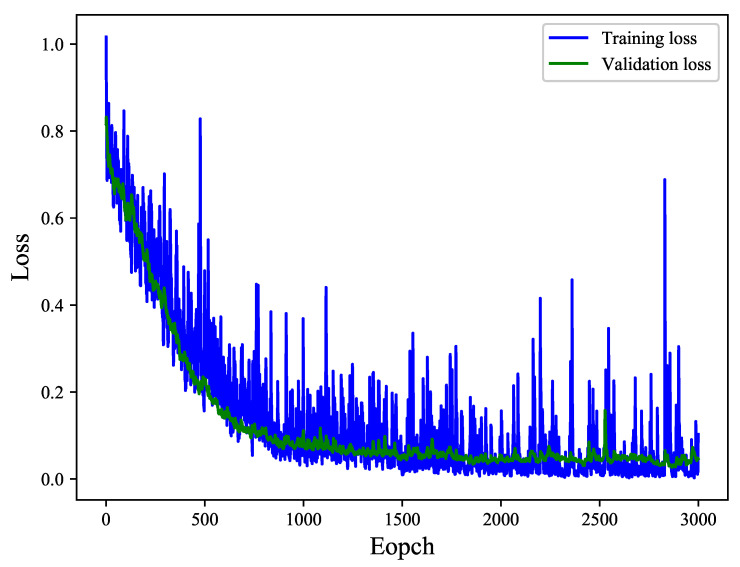
Training and validation loss curve of the model as a function of epochs.

In this paper, 16,000 micrographs of 100 groups of samples are collected on the z-axis at 1 μm intervals and divided randomly as 80% for training and 20% for testing. The cross-entropy is used between the predicted class labels and the true class labels as the loss function for training, and the gradient descent optimizer is used with backpropagation to update the network weights. To calculate the gradient of the loss function per epoch, a small batch size of 20 is used. FSR model is trained from scratch and the weights of the network initialized following the Glorot uniform initialization technique [30]. The learning rate decay algorithm adopts exponential decay which factor is set to 0.94. In addition, five samples are randomly selected from the test set as the verification set in each round of training. After 3000 epochs, the metrics used for evaluation of the model’s performance are accuracy, precision, recall, and F-score, respectively. The training and validation loss per epoch can be observed in Figure 6. It can be seen that the training loss and the testing loss for both methods reach stable values after 2000 epochs, no overfitting is observed. For this FSR model, it is obtained with an accuracy of 97.92, a precision of 98.89, a recall of 96.93, and an F-score of 97.93, respectively.

In the present study, all training and testing experiments of models are conducted on a Windows system platform that has an Intel Core (TM) 3.4 GHz processor with 16 GB of RAM and an NVIDIA GeForce 1080Ti GPU.

The number of output layers of classic ResNet101 and VGG16 is changed to 2 as the focus state recognition model and they are compared with our model. They are trained and evaluated under the same conditions as FSR. The statistics of focus state recognition models based on DCNNs are shown in Table 1. We count the single number of training times to compare the time cost of training different models. In the last column, we report running time in milliseconds (ms) on the CPU. The focus state recognition models based on ResNet101 and VGG16 cannot be run on our existing microscopically controlled equipment due to complex calculations. Results of the best performing focus state recognition models are in bold. It can be seen from Table 1 that our approach works well on each of the four indicators.

### 3.2. Effects of ESMS

The parameters of the ESMS algorithm in the experiment are set as follows: The scaling factor is 0.9; the crossover probability is 0.8; the population *M* is 6; the maximum number of searches *T* is 15; the constraint lower: −15.520 cm, upper: −15.680 cm; the lens accuracy ε is 0.001 cm.

**Figure 7:**
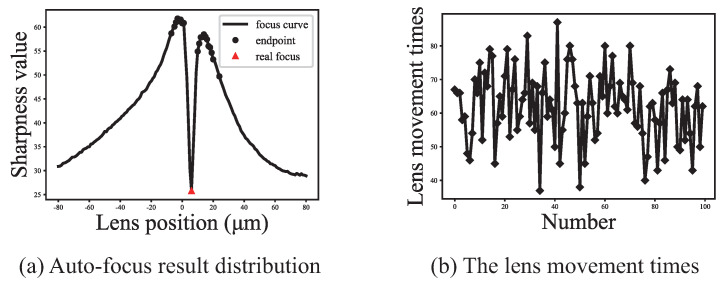
Auto-focus with traversal search.

**Figure 8:**
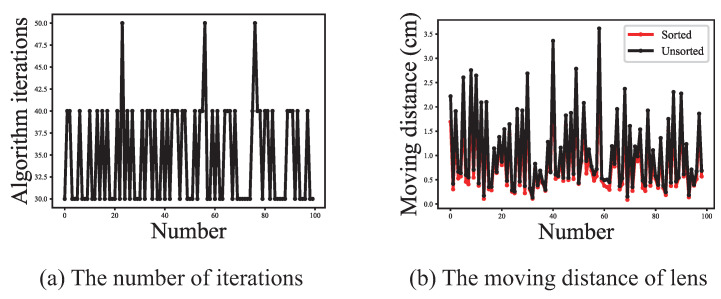
Sorting and memory functions of ESMS.

The purpose of this section is to analyze the effectiveness of ESMS separately, so the region search algorithm only considers the traversal search. Figure 7a shows the distribution of the endpoint positions and the real focus positions finally obtained by auto-focus with traversal search. It is easily observed that ESMS can effectively obtain the endpoint positions. Figure 7b shows the movement times of the motor. The initial number of iterations of one-round of ESMS is 15, and the final iterations number of auto-focus is shown in Figure 8a. It can be seen that usually necessary to perform ESMS 2 to 3 times to obtain the correct focus position, and also further proves the effectiveness of the squeeze adjustment algorithm. Comparing Figure 7b with Figure 8a, we can see that the actual evaluation times of the population in the ESMS algorithm are far lower than the set times of the theory. It verifies that establishing the historical evaluation of DE-best can effectively reduce the number of movements of the motor. As shown in Figure 8b, the total moving distance of the motor is significantly reduced after each sorting for the population of each generation.

### 3.3. Performance of Auto-Focus Method with Various Region Search Algorithms

**Figure 9:**
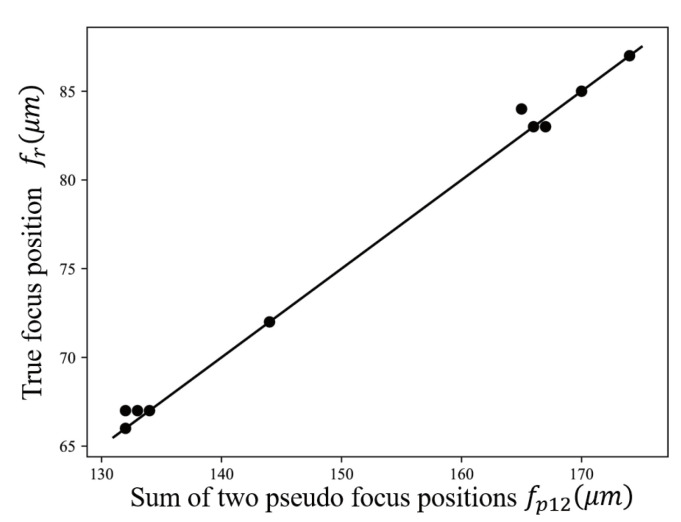
Distribution of the sum of two pseudo focus positions and real focus position.

**Figure 10:**
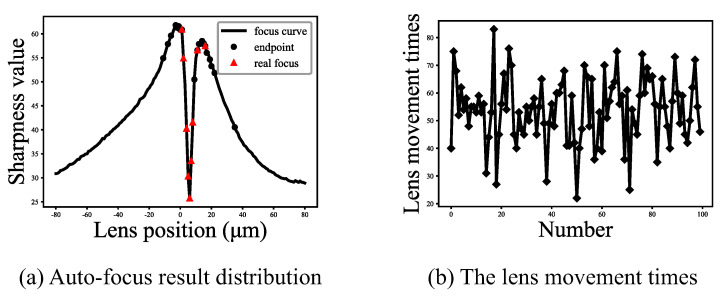
The actual number of move of microscope lens with different region search algorithms.

The distribution of the sum of two pseudo focus positions $f_{p12}$ and real focus position $f_{r}$ is shown in Figure 9. The calculated value of the Pearson correlation coefficient is 0.997. Therefore, in the region search algorithm, either traversal search or mathematical prediction can be selected. The value of parameter *k* in Formula (5) is 0.5 obtained by using the least square method. The endpoint positions and real focus positions obtained by auto-focus with mathematical prediction are shown in Figure 10a. The endpoint positions and real focus positions obtained by the result of auto-focus with traversal search are shown in Figure 7a. The auto-focus with traversal search always obtains the minimum value between two endpoint positions, and has higher accuracy than auto-focus with mathematical prediction. Generally, the area of 3 μm near-ideal focus position is set as the effective focus area. In this experiment, the success rates of auto-focus with traversal search and auto-focus with mathematical prediction are 100% and 95% respectively. In Figure 10b, it can be seen that the average number of moving lens has been reduced from 61.1 to 53.8, where has indicated that the auto-focus speed of auto-focus with mathematical prediction is faster than auto-focus with traversal search. For some cases where the requirement of focus accuracy is not high, and the requirement of speed is strict, the auto-focus with mathematical prediction can be used.

## 4. Conclusions

In this paper, a novel auto-focus method of the microscope was developed for the surface structure of transparent materials under transmission illumination. In this auto-focus task, two distinct focus states would exist in the focusing process while the real focus position is actually located between the two states with a local minimal sharpness value in the focus curve. Thus, it is quite different from the existing auto-focus problems with only one focus state. First, a DCNN model (FSR) has been established to learn microscopic image features adaptively through the convolution layer to classify the focus states. Then, an evolutionary computing algorithm (ESMS) has been designed to get the positions of the two focus states. Finally, traversal search and mathematical prediction have been given to obtain the real focus position between the two focus states. By the experiments of data sets collected by an actual microscope experimental platform, it has been shown that the FSR can achieve an F-score of 97.88%; the success rates of auto-focus with traversal search and auto-focus with mathematical prediction are 95% and 100%, respectively; and our method is capable of solving this particular auto-focus problem.

In future work, the scalability of our method will be verified on different microscope imaging systems and some work will be carried out on evolutionary computation and image classification for further improvement.

## Figures and Tables

**Table 1:** Focus State Recognition Model based on DCNN.

Model	F-Score	Params	Training Time	CPU Testing Time
FSR(ours)	**97.93%**	**4.3M**	**0.3 h**	**190 ms**
VGG16	90.3%	512M	2.1 h	-
ResNet101	97.3%	591M	3.2 h	-

## Data Availability

The data used to support the findings of this study are available from the corresponding author upon request.
